# Acute Intermittent Porphyria: A Review and Rehabilitation Perspective

**DOI:** 10.7759/cureus.44260

**Published:** 2023-08-28

**Authors:** Adriana Valbuena Valecillos, Puja Yatham, Matison Alderman, Lauren Shapiro, Eduard Tiozzo, Joslyn Gober

**Affiliations:** 1 Department of Physical Medicine and Rehabilitation, University of Miami Miller School of Medicine/Jackson Memorial Hospital, Miami, USA; 2 Department of Medicine, Herbert Wertheim College of Medicine, Miami, USA

**Keywords:** rare diseases, functional status, rehabilitation, paralysis, acute intermittent porphyria

## Abstract

Acute intermittent porphyria (AIP) is an uncommon metabolic disease that impacts multiple organs and can manifest in many ways. It is often misdiagnosed due to its nonspecific symptoms. Neurovisceral signs and symptoms should alert physicians to consider AIP in the differential after excluding more common causes. Identifying the underlying cause is critical in preventing acute attacks, and trigger avoidance is the optimal approach to managing AIP. Medications that are contraindicated should be reviewed thoroughly. Prompt intravenous hematin administration is the primary treatment for acute attacks, and additional pharmacological therapies may be necessary to treat concurrent symptoms. A severe neurological manifestation of AIP is flaccid paralysis or severe motor weakness, which can develop into total quadriplegia and respiratory insufficiency. A comprehensive rehabilitation program is an integral aspect of the treatment plan. Since the incidence of this disease is low, functional prognosis is not well-known. As a result, it is challenging to determine the most appropriate structure, intensity, and duration of rehabilitation therapy. By extending the treatment plan, individuals with tetraplegia due to AIP can continue to make functional gains years after the onset of weakness. Understanding the disease’s functional prognosis will aid in coordinating resources and improving healthcare expenditures.

## Introduction and background

Porphyrias are a group of disorders that primarily affect the nervous system or the skin, according to the National Organization of Rare Disorders (NORD), occurring when there is an abnormality in the pathway for the production of heme [1]. Heme is an essential constituent of hemoglobin, myoglobin, catalase, peroxidase, respiratory, and P450 liver cytochromes [2]. These disorders arise because of an enzyme deficiency in the eight-step heme synthesis pathway, leading to a buildup of toxic intermediates called porphyrins and porphyrin precursors, with the latter being neurotoxic.

According to the consensus of the International Acute Porphyria expert panel, acute porphyrias are classified as acute intermittent porphyria (AIP), variegate porphyria (VP), or hereditary coproporphyria (HCP) [3]. Acute porphyrias affects the central (CNS), peripheral (PNS), and autonomic nervous systems and usually presents with symptoms including abdominal discomfort, mental status changes, pain, and weakness. AIP is commonly associated with neurological disease and will thus be the focus of this manuscript [4,5].

The goal of this paper is to highlight how AIP can present with various neurological manifestations, including flaccid paralysis. Treatment is highly effective, and given the uncertainty regarding a temporal relationship between treatment and overall morbidity, it is best to identify the cause and initiate treatment promptly. Currently, there is extraordinarily little research specifically on the rehabilitation of patients with flaccid paralysis due to AIP. As a result, it is challenging to predict the function, outcomes, and future needs of these patients, including, but not limited to, the equipment and support they will need once discharged from the hospital setting.

Epidemiology

Porphyrias are genetically inherited; the majority are autosomal dominant. Due to the various phenotypes, prevalence can be difficult to evaluate, and calculations are usually underestimated [1]. According to the Rare Disease Clinical Research Network, the combined prevalence of acute porphyrias is five cases per 100,000 persons [6]. For AIP specifically, the estimated prevalence of likely pathogenic mutations is about 560 million [7]. The clinical expression of porphyrias is also exceptionally variable, as 90% of heterozygotes remain asymptomatic throughout life. In those that are clinically symptomatic, acute attacks are usually more common in females and manifest after puberty [2,8].

More than 400 variants in the hydroxymethylbilane synthase (*HMBS*) gene causing AIP have been listed in the Human Gene Database [9]. The prevalence of *HMBS* mutations in Caucasians is estimated to be one in 7,000 [7]. However, acute porphyric attacks occur only in 1% of the estimated prevalence, emphasizing the importance of genetic modifiers and environmental exposures [9,10]. A three-year prospective study of patients diagnosed with AIP in 11 European countries showed that the annual incidence of symptomatic AIP was 0.13 per million. AIP was the most common porphyria, with a prevalence of 5-10 per million, and the recurrence rate of AIP was between 3% and 5% [11]. Two studies conducted in France estimated the prevalence of mutations in the *HMBS* gene to be one in 1,675 and one in 1,299, respectively [12,13]. A prospective study on the molecular epidemiology of porphobilinogen (*PBG*) deaminase gene defects in AIP showed that *PBG* deaminase gene mutations were found in 109 families out of 121 unrelated French Caucasian AIP families [14]. The prevalence of AIP was noted to be three times higher in some regions of Spain with a high frequency of *HMBS* founder mutation [15]. The results of this study suggested that CYP2D6*4 and CYP2D6*5 alleles may be protective factors against acute attacks, and *CYP2D6* may constitute a penetrance-modifying gene [15].

Pathophysiology

**Figure 1:**
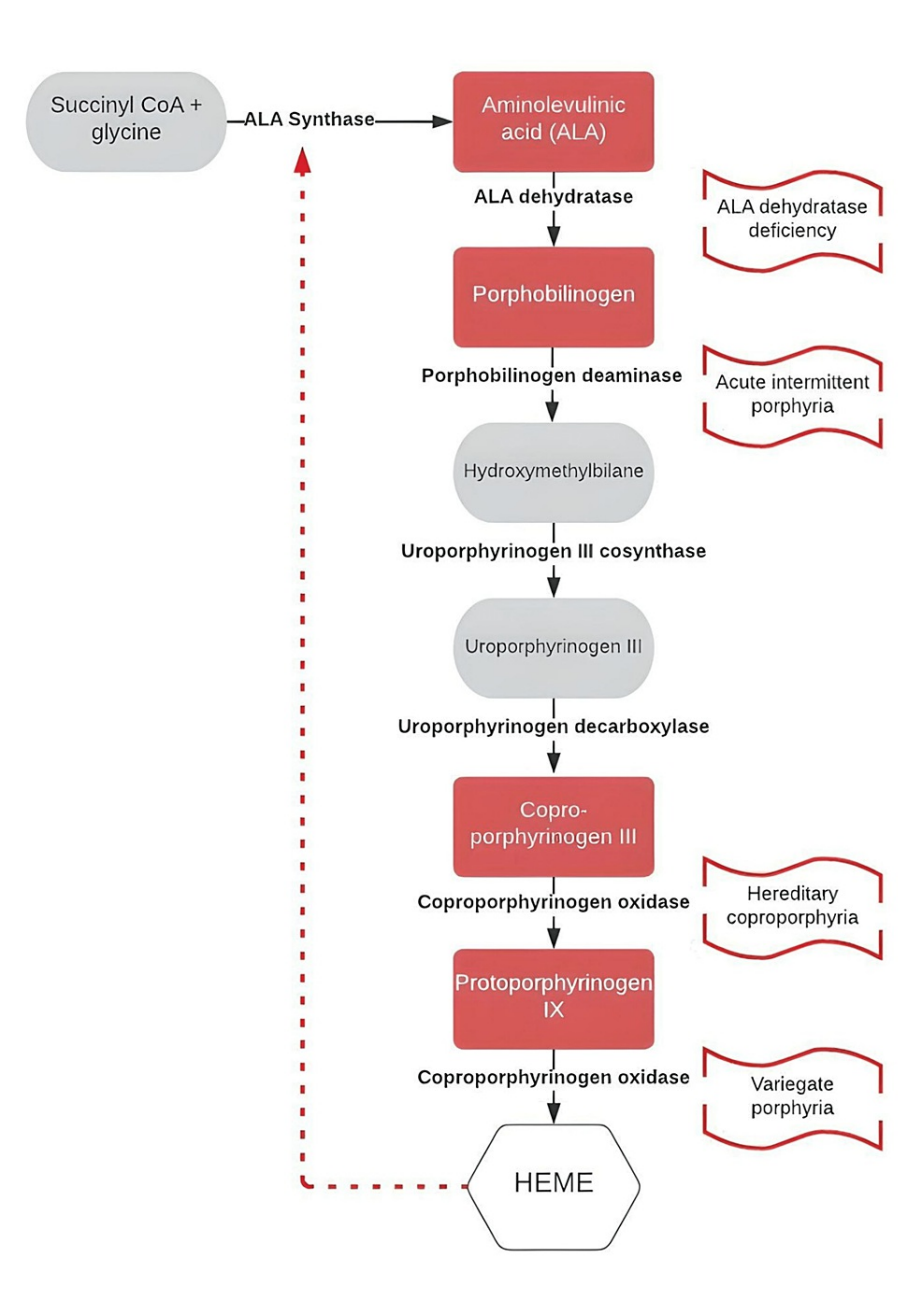
Heme synthesis pathway with a focus on the enzyme deficiencies causing acute porphyrias ALA: aminolevulinic acid

Porphyrias are disorders of heme metabolism, each the result of a dysfunctional enzyme involved in the eight-step synthesis of a heme molecule (Figure 1). Heme synthesis takes place in all cells and begins in the mitochondria. Normally, *PBG* catalyzes to hydroxymethylbilane by the enzyme PBG deaminase, which leads to excessive amounts of *PBG*. However, a defect in this process causes AIP.

The enzyme malfunction in the heme pathway leads to an accumulation of the upstream porphyrin precursors, resulting in the accumulation of toxic intermediates, which result in a variety of signs and symptoms. The mechanism of how the porphyrin precursors cause both central and peripheral nervous system dysfunction is poorly understood, and given the range of clinical presentations, it is highly likely that there are various mechanisms. The two main hypotheses attribute the neurological damage to the toxicity of porphyrin precursors and the overall deficiency of heme. The porphyrin precursors, aminolevulinic acid (ALA) and porphobilinogen (PBG), which are the first and second intermediates of the pathway, are thought to be neurotoxic [4]. ALA is able to cross the blood-brain barrier and is known to be toxic to glial and neuronal cells in culture [16]. One hypothesis suggests that the similar structures of ALA and gamma-aminobutyric acid (GABA) result in impaired GABA function secondary to the buildup of ALA [17]. The heme deficiency theory is easily supported by the resolution of the acute attack with IV heme treatment. The deficiency leads to reduced mitochondrial cytochromes and other important hemoproteins required for the electron transport, causing several downstream events such as impaired oxidative phosphorylation and reduced ATP production in neurons and glial cells, impaired Na+/K+ pump function for axonal transport, and impairment of other ATP-mediated signaling in the nervous system [18].

A review of pathology studies from 35 patients with AIP included histological findings of the following: neurogenic atrophy, segmental demyelination, axonal swelling and fragmentation, spinal root demyelination and axonal loss, dorsal root ganglia degeneration, anterior horn cell degeneration, intermediolateral cell column degeneration, and posterior column degeneration [19]. Given the lack of significant central nervous system findings in most case reports, the flaccid paralysis is likely not a result of a neuroinflammatory process in the CNS and the proximal nerves [20].

## Review

To illustrate a case of a severe neurological presentation of AIP, we present a clinical vignette. A written consent was obtained from the patient to share her story.

A 21-year-old female presented with persistent severe abdominal pain. She had a history of chronic intermittent abdominal pain attributed to an ovarian cyst, for which she underwent laparoscopic resection and left ureterolysis and was discharged home with oral contraceptives. However, the abdominal pain never resolved, and she required multiple visits to the emergency room with inconclusive workups. She ultimately presented with this abdominal pain in addition to new-onset tonic-clonic seizures, sudden ascending paralysis, and respiratory failure requiring mechanical ventilation.

The initial presumptive diagnosis was Guillain-Barré syndrome due to the clinical presentation of respiratory failure and ascending paralysis, along with a nerve conduction study and electromyography (NCS/EMG) that showed evidence of diffuse axonal motor polyneuropathy. She subsequently completed a five-day course of intravenous immunoglobulin (IVIG) without significant improvement. At that time, the medical board of the local institution was concerned for porphyria and decided to transfer her to a US tertiary referral hospital for further workup and treatment.

On admission to this hospital, she required continuation of mechanical ventilation; her vital signs were significant for persistent tachycardia and hypertension. The physical examination was remarkable for absent motor activation in the proximal upper and lower extremities, including bilateral biceps, triceps, wrist flexors and extensors, hips flexors and extensors, and knees flexor and extensors. She showed partial antigravity motor activation distally, including finger flexion, ankle dorsiflexion, plantar flexion, inversion, and eversion. The reflexes were absent throughout. Sensation to light touch and vibration was intact in all extremities. The laboratory findings were significant for elevations of serum aminotransferases, hyponatremia, and hypomagnesemia. Hepatitis markers were negative, and abdominal ultrasound was normal. A lumbar puncture was normal. The pregnancy test was negative.

After excluding multiple diagnoses, the quick neurological deterioration, and the high clinical suspicion for an acute porphyria attack, it was consensus to start the treatment for AIP empirically. Ultimately, urine and laboratory results for porphyria workup revealed a total porphyrin level of 25.6 mcg/L (normal range (NR): 1.0-5.6 mcg/L), porphobilinogen urine of 184.921 mg/gCre/12 hour (NR: <0.22 mg/gCre/12 hour), uroporphyrin of 18 mcg/L (NR: <0.2 mcg/L), heptacarboxyporphyrin of 1.9 mcg/L (NR: <0.2 mcg/L), hexacarboxyporphyrin of 0.7 mcg/L (NR: <0.3 mcg/L), pentacarboxyporphyrin of 1.1 mcg/L (NR: <0.4 mcg/L), coproporphyrin of 2.6 mcg/L (NR: <0.8 mcg/L), and protoporphyrin of 1.3 mcg/L (NR: 0.4-4.8 mcg/L).

This chemical profile associated with the clinical presentation confirmed the diagnosis of acute porphyria, which was subsequently confirmed with genetic testing. Repeat NCS/EMG at this time showed subacute to chronic severe diffuse motor axonal polyneuropathy. Seven days after hemin infusions, porphobilinogen decreased by almost sixfold (32.380 mcg/L). After two weeks in our intensive care unit, the patient was successfully extubated and was able to speak with severe dysphonia due to bilateral vocal cord paresis. A feeding tube was placed for pharyngeal dysphagia and maintained due to poor calorie intake. She was able to tolerate out-of-bed activities, although challenged by orthostatic hypotension.

She was later transferred to an acute inpatient rehabilitation hospital, where she was managed by a multidisciplinary team including nursing, physical, occupational, speech therapy, and recreational therapy, as well as neuropsychology and dietician under the medical supervision of a rehabilitation physician. She continued receiving hemin infusions weekly while undergoing the intensive rehabilitation program of three hours of therapy every day (Table 1). Following a 21-day-long stay, she could activate her upper extremities with gravity eliminated and bilateral lower extremities against gravity without resistance. Family training was completed, and she was discharged home with the proper equipment and an outpatient rehabilitation program.

**Table 1:** Inpatient rehabilitation program ROM: range of motion, VS: vital sign

Discipline	Frequency	Intervention	Duration
Physical therapy	6 sessions per week (60 minutes)	Passive and active ROM of the lower extremities, muscle strengthening exercises of the lower extremities, static balance training, dynamic balance training, functional electrical stimulation in lower extremities muscle, orthotic evaluation (foot drop orthosis), gait training with partial weight support (zero gravity)	3 weeks
Occupational therapy	6 sessions per week (60 minutes)	Passive and active ROM of the upper extremities, muscle strengthening exercises of the upper extremities, stance balance training, dynamic balance training, functional electrical stimulation in the upper extremities muscle, orthotic evaluation (i.e., wrist drop orthosis)	3 weeks
Speech therapy	6 sessions per week (60 minutes)	Phonation vocal exercises, evaluation of swallowing ability, instruction in dysphagia compensatory strategies	3 weeks
Recreational therapy	2 sessions per week (45 minutes-1 hour)	Promote leisure skills and social interaction	3 weeks
Dietitian	2 sessions per week	Monitoring calorie intake, education about dietary triggers	3 weeks
Psychology	2 sessions per week (45 minutes-1 hour)	Emotional support for patient and family, facilitate adjustment to disability, coping mechanism for pain management	3 weeks
Nursing	24 hours a day	VS monitor, implement preventive measures to prevent skin breakdown	3 weeks
Respiratory	6 sessions per week	Respiratory therapy, assisted cough training	3 weeks
Physiatrist	24 hours a day	Management and treatment of abnormal VS and electrolytes, monitor proper nutrition and adjusting feeding, pain management, bowel and bladder management, leading rehabilitation team	3 weeks

She continued receiving hemin infusions and rehabilitation therapies on an outpatient basis for almost two years. Her functional abilities significantly improved. From being entirely dependent, she was able to reach complete independence in all activities of daily living (ADLs) and mobility. She was able to walk, run, and dance without the use of any assistive device.

The preceding vignette describes a severe neurological presentation of respiratory failure and flaccid quadriparesis due to AIP. Porphyrias are known as “mimickers” of more common neurological or psychiatric diseases [21]. The classical triad of abdominal pain, psychosis, and neuropathy is a well-known association with porphyrias [21]. However, the spectrum and unpredictable presentations with no associated family history make the diagnosis difficult.

Given how rare acute porphyrias are, as well as their nonspecific symptoms, they are often not on the differential diagnosis when persons present with diffuse muscle weakness. Porphyria should be suspected after ruling out more common causes in patients presenting with neurovisceral signs and symptoms [22,23]. Although porphyrias are rare, it is essential to consider them earlier in the patient’s workup as irreversible complications can result from delayed treatment, leading to significant impairments of multiple body systems and even death [4].

The best management of these disorders is preventing acute attacks via identifying and avoiding triggers (Table 2). Porphyric neurovisceral attacks are often triggered by stress, alcohol, smoking, fasting or severe carbohydrate-restrictive diets, drugs (especially cytochrome P450-inducing agents), infectious diseases, or cyclical hormonal changes [24]. Identifying the inciting factor is critical in the prevention of further progression of the disease. However, some attacks may not have an identifiable trigger.

**Table 2:** Common triggers of porphyric attacks NSAIDs: nonsteroidal anti-inflammatory drugs, UV: ultraviolet

Triggers	Examples
Medications	Barbiturates (e.g., phenobarbital, primidone), certain antibiotics (e.g., sulfonamides, fluoroquinolones), estrogens (hormonal medications), antiepileptic drugs (e.g., carbamazepine, valproate), NSAIDs
Diet and fasting	Low-calorie or extreme diets, prolonged fasting or starvation, certain food triggers (e.g., alcohol, high-protein foods)
Hormonal changes	Hormonal fluctuations during menstrual cycles, hormonal changes during pregnancy, hormone replacement therapy
Stress and emotional factors	Physical or emotional stress, anxiety or trauma, emotional upheaval or distress
Environmental factors	Infections or other illnesses, exposure to certain chemicals or toxins, excessive exposure to sunlight or UV light, smoking, including the use of marijuana

Multiple medications are contraindicated for patients with porphyria. Therefore, it is important to review a patient’s medication list [23]. For example, severe porphyria attacks have been associated with barbiturates and sulfonamides, in addition to many other drugs that are classified as potentially porphyrinogenic due to a lack of safety in patients with porphyria [25]. A list of medications evaluated by their safety for patients with porphyria can be found in the European Drug Database for Acute Porphyrias or the American Porphyria Foundation drug database [26].

The female sex hormones, especially estradiol, are often known inducers of heme synthesis, increasing risks for porphyria attacks. In fact, pregnancy itself may incite an attack [27]. General anesthesia is also known to be related to triggering acute porphyria attacks [28]. Thus, knowing if a patient is a carrier of the trait is also important for the outcome of surgery [29]. Management of acute intermittent porphyria involves education and genetic screening of family members, including the patient’s parents, siblings, and children [30].

With avoiding or discontinuing triggering agents, early pharmacological intervention of acute intermittent porphyria is essential to provide rapid treatment of symptoms and prevention of complications. The first line of treatment is the prompt administration of intravenous hematin [31]. These injections limit the body’s production of porphyrins. Intravenous glucose can be used for mild attacks or when there is a delay in the accessibility of hemin in order to maintain an adequate intake of carbohydrates [31]. In addition, treatment with erythropoietin can reduce ALA, PBG, and porphyrin levels and reduce the severity of attacks [31]. In 2019, givosiran was FDA-approved for the treatment of acute hepatic porphyrias. It has shown benefits in patients with recurrent attacks [32].

Additionally, pharmacological interventions may be required to treat associated symptoms. One of the most common challenges while treating a patient with acute intermittent porphyria is the severity of pain and multiple medication contraindications. Severe paroxysmal abdominal pain and nervous system presentations are usually the most frequent clinical manifestations of an acute attack of AIP [33]. There have also been reports of low back and leg pain [34]. In patients with a mild presentation, paracetamol (or acetaminophen) and anti-inflammatories have been shown to help [25]. Several opioid medications are safe in this population, including morphine, pethidine, oxycodone, tramadol, and fentanyl. In fact, opioid analgesia pumps are often required in the acute phase of an attack before transitioning to oral medications [26]. Gabapentin, which is also safe to use according to the database for acute porphyria, could be used as an adjunctive therapy for neuropathic pain, neuropsychiatry presentations, and seizure management [35]. Other neuropathic pain agents that are considered safe in the treatment of acute porphyrias include amitriptyline, duloxetine, and pregabalin [26].

Patients with acute intermittent porphyria could be misperceived as drug-seeking due to the severity of their pain without a precise diagnosis. These populations are at risk of chronic pain issues and opiate dependency. Therefore, a multidisciplinary team (including physicians, nurses, psychologists, and therapists) should be involved to address the complex nature of this disease.

Nausea and vomiting are also common presentations in AIP attacks, whether due to pain or abdominal etiology. Patients may present with an acute abdomen associated with nausea and vomiting, which is known as an acute neurovisceral attack [31]. Prochlorperazine and ondansetron are considered safe options for nausea and vomiting [23]. Olanzapine and lorazepam can also be used as alternative treatments. Metoclopramide had been associated with the exacerbation of porphyria attacks. However, it has more recently been reported to be helpful without complications [25]. The use of domperidone is limited as it may interact with opioids and other drugs, increasing the risk of arrhythmias and adverse cardiac reactions [25].

Patients with AIP can present with constipation and paralytic ileus. This may be due to multiple factors, including the underlying primary neurological etiology and associated side effects of opioid use [34]. For management, a bowel program should be established early in the presentation. Senna and bisacodyl are considered safe treatment options [26].

Due to the neurological presentation of this disease and the risk of neurogenic bladder, monitoring of urinary output is required to ensure complete bladder emptying. Post-void residual volumes (PVR) should be monitored, and if high, the patient should be started on a bladder program with a Foley catheter or straight catheterizations. Additionally, urodynamic studies may be beneficial to help classify bladder function.

Neurological complications may include both central nervous system (CNS) and peripheral nervous system (PNS) signs and symptoms [31,36]. The initial neurological presentations of AIP include altered mental status (AMS), headache, seizure, autonomic dysfunction, quadriparesis, and visual disturbance [36]. It is also known to have brain MRI findings suggestive of posterior reversible encephalopathy syndrome (PRES) [31,33,36]. The literature suggests including AIP as a differential diagnosis when severe paroxysmal unexplained abdominal pain is associated with PRES in brain imaging [34].

While PRES is nonspecific and not pathognomonic for AIP, it is often seen when individuals present with seizures and hypertension. Generalized tonic-clonic seizures and partial seizures are the types of seizures described in this pathology [34]. Seizure management is also challenging in AIP because several commonly used medications are hazardous in patients with porphyria [30]. For example, it is recommended to avoid carbamazepine, phenytoin, and valproic acid due to their liver metabolism and potential hepatotoxicity, as these patients may eventually require a liver transplant [30].

Neuropathy occurs in up to 40% of acute porphyric attacks and can progress to complete quadriplegia and respiratory insufficiency, requiring ventilator support [22]. Sensation abnormalities are usually described as numbness or pain and present in a “glove and stocking” distribution [37]. Sensory examination, including vibration and proprioception, is typically normal, but reflexes are usually hypoactive or absent [24].

One of the most disabling and concerning neurological presentations of AIP is motor weakness or flaccid paralysis. Flaccid paralysis is a clinical syndrome characterized by a rapid onset of weakness, commonly resulting from a lower motor neuron lesion, including weakness of the respiratory and pharyngeal muscles [38]. The severity of the weakness from AIP can vary from mild to severe quadriparesis and can be associated with respiratory failure [34]. Clinically, it often presents as diffuse flaccidity, and axonal damage is typically more evident in proximal muscles, particularly in the upper extremities [31]. Electromyography and nerve conduction studies are significant for axonal polyneuropathy, with fibrillation potentials and decreased motor unit recruitment. Cranial nerve dysfunction has also been reported, most commonly affecting the facial and vagus nerves [31]. For patients presenting with severe motor polyneuropathy associated with bulbar palsy, mechanical ventilation is often required for respiratory support [34]. It is important to differentiate porphyria neuropathy from others, such as Guillain-Barré syndrome (GBS) and lead toxicity, as prompt treatment can significantly impact long-term outcomes.

There is an increased prevalence of psychiatric disorders among individuals with porphyria. These include anxiety, depression, adjustment disorder, behavioral changes, and substance use disorder [37]. Fluoxetine, paroxetine, amitriptyline, haloperidol, and lithium are considered safe treatment options. However, trazodone, quetiapine, and ziprasidone are contraindicated [26].

Abnormal vital signs are commonly seen in AIP as well, particularly hypertension and tachycardia. This seems to be related to the autonomic nervous system with overactivity of the sympathetic nervous system specifically [25,34]. It is usually treated with beta-blockers, angiotensin-converting enzyme inhibitors, and calcium channel blockers, with diltiazem preferred over nifedipine, which is porphyrogenic in model systems [21].

Electrolyte abnormalities can be seen in AIP. Hyponatremia is usually present early in the acute attack, and it is thought to be related to the syndrome of inappropriate antidiuretic hormone secretion or excessive losses. Such metabolic derangements could contribute to the neurological manifestations [39]. Hypomagnesemia can also be seen in the acute flare, and it is thought to be a result of hypothalamic involvement or excessive loss in feces or urine [25]. As such, close monitoring and correction of the electrolyte imbalance are important.

Recurrent attacks are possible with a rate of 3%-5%, largely depending on how well an individual follows the guidelines and prevents triggers [11]. Decreased quality of life and functional decline are often associated with recurrent attacks, which can vary from months to years after the initial presentation [30]. Prophylactic heme infusion is helpful in some cases [30]. However, it is less commonly used due to the availability of givosiran. Givosiran has been shown to reduce the number of porphyria attacks, decrease the need for hemin use, and improve associated pain. It is generally well tolerated and is provided as a monthly subcutaneous injection [32,40]. The need for liver transplants has declined in this population as a result of effective prophylactic therapies [23].

Rehabilitation strategies and outcomes

Due to its variable clinical presentation and multiorgan involvement, a comprehensive rehabilitation program can play an integral role in the treatment protocol for AIP. It is important not only for severe motor weakness but also to prevent the high risk of complications associated with prolonged bed rest, such as pressure injuries, deep vein thrombosis, and pneumonia.

Bonnefoy Mirralles et al. discussed a case report of a 43-year-old patient with dysphagia, dysphonia, severe flaccid quadriparesis, and poor trunk control, who was unable to sit unsupported, stand up, or walk, making her totally dependent for all activities of daily living (ADLs) due to AIP. She was enrolled in an inpatient comprehensive rehabilitation program for 14 months, including physical, occupational, speech, and respiratory therapy, as well as psychological support, followed by an outpatient program. The Berg scale and functional independence measure (FIM) assessment were the functional metrics used to monitor outcomes. At her two-year follow-up, she achieved complete independence with mobility and ADLs; she just needed some assistance tying her shoelaces and buttoning and buckling articles of clothing [41].

The prognosis for regaining mobility and walking again after an episode of quadriparesis due to AIP varies among individuals. Some patients may experience a partial or complete recovery of motor function over time with appropriate treatment and rehabilitation interventions [41]. However, the extent of recovery can be influenced by factors such as the duration and severity of quadriparesis, any nerve damage or muscle weakness, and individual variations in the disease course [20,41].

A rehabilitation program, including a physiatrist, physical therapist, occupational therapist, speech therapist, nutritionist, psychologist, and other supportive interventions, is crucial in optimizing functional recovery and maximizing independence. These therapies focus on strengthening muscles, improving coordination, enhancing mobility, family training, and facilitating adaptive strategies to manage residual impairments, ensure safe discharge, and foster integration into the community.

The nutritionist aims to support overall health and minimize dietary triggers that may induce porphyria attacks. Dietary recommendations for individuals with AIP include maintaining stable carbohydrate levels. Consuming a diet that contains moderate to high carbohydrates (e.g., 60%-70% of total calories) from complex sources such as whole grains, legumes, fruits, and vegetables is recommended. Avoiding extreme or rapid fluctuations in carbohydrate intake is crucial. Additionally, staying well-hydrated is essential for individuals with AIP. Protein intake should be adequate to support normal body functions and promote tissue repair. However, excessive protein intake may contribute to an accumulation of porphyrins. Consuming a moderate amount of protein from sources such as lean meats, poultry, fish, eggs, dairy products, legumes, and tofu is generally advised. Alcohol consumption should be strictly avoided, as it can trigger porphyria attacks, and so can certain spices and excessive amounts of caffeine [21].

Orthoses and durable medical equipment (DME), including adaptive equipment, can play a supportive role in managing the neurological presentation of AIP by addressing specific symptoms and promoting functional independence. Patients with AIP may experience muscle weakness or peripheral neuropathy, leading to instability and pain; therefore, evaluation for bracing, assistive devices, and DME should be completed by the multidisciplinary team. These may include ankle-foot orthotics (AFOs), wrist-hand orthotics, and assistive devices for activities of daily living (ADLs) such as reachers, dressing aids, adaptive utensils, and devices designed to assist with bathing or toileting, as well as walkers, wheelchairs, or other mobility aids. Additionally, ongoing evaluation and follow-up are crucial to ensure optimal fit, function, and effectiveness of all the prescribed orthotics or DME. In rare cases, neurological symptoms associated with AIP may affect speech or impair communication. Augmentative and alternative communication (AAC) devices can assist individuals in expressing themselves effectively, allowing for improved social interaction and participation.

Understanding the disease’s functional prognosis ultimately helps better coordinate the resources and improve healthcare expenses. For example, in the United States, the average length of stay for an inpatient rehabilitation program is 2-3 weeks [42]. In our experience, by that time, patients with flaccid quadriplegia due to porphyria are often still dependent on most, if not all, daily activities, and they require costly custom-made equipment for essential mobility. However, with the proper interventions and rehabilitation program, this equipment may not be necessary several months to a year after the initial presentation. As such, extending the therapy program for this patient population may be beneficial as they are expected to make functional gains, even two years after the initial event.

Intensive inpatient rehabilitation programs are often necessary in the early part of one’s recovery following an AIP attack resulting in quadriplegia, but it is important they are viewed as only the first stop in one’s long journey along the rehabilitation continuum of care. In the United States, the average length of stay in inpatient rehabilitation is only approximately two weeks long. Thus, in our experience, affected patients often return home continuing to require assistance with mobility and self-care and with equipment that is rented rather than customized for their needs. It is important that rehabilitation professionals provide appropriate training to their caregivers so that they may best assist them in the home environment. Moreover, members of the rehabilitation team should reinforce the long-term potential for improvement, with the likelihood of regaining of one’s functional independence.

## Conclusions

AIP affects multiple organ systems and may present in a variety of manifestations. If treated on time with the appropriate interventions, patients have the potential for full recovery. Therefore, clinicians should be familiar with this rare condition to avoid diagnosis and treatment delay, preventing debilitating and potentially life-threatening consequences.

Rehabilitation programs, including both an acute inpatient rehabilitation program and a transition to outpatient services, can significantly reduce the disability associated with the neurological presentations of porphyria. Due to the limited literature regarding the expected functional prognosis for these patients, the decision for the appropriate DMEs and the structure, intensity, and length of the rehabilitation program can be challenging. As such, discovering ways that can help predict potential functional recovery is essential to deciding the most appropriate medical resources.

## References

[1] Anderson KE. Porphyrias. NORD (2023). Porphyrias. https://rarediseases.org/rare-diseases/porphyria/.

[2] Lin CS, Krishnan AV, Lee MJ (2008). Nerve function and dysfunction in acute intermittent porphyria. Brain.

[3] Stein PE, Edel Y, Mansour R, Mustafa RA, Sandberg S (2023). Key terms and definitions in acute porphyrias: results of an international Delphi consensus led by the European porphyria network. J Inherit Metab Dis.

[4] Gerischer LM, Scheibe F, Nümann A, Köhnlein M, Stölzel U, Meisel A (2021). Acute porphyrias - a neurological perspective. Brain Behav.

[5] Alqwaifly M, Bril V, Dodig D (2019). Acute intermittent porphyria: a report of 3 cases with neuropathy. Case Rep Neurol.

[6] Spiritos Z, Salvador S, Mosquera D, Wilder J (2019). Acute intermittent porphyria: current perspectives and case presentation. Ther Clin Risk Manag.

[7] Chen B, Solis-Villa C, Hakenberg J (2016). Acute intermittent porphyria: predicted pathogenicity of HMBS variants indicates extremely low penetrance of the autosomal dominant disease. Hum Mutat.

[8] Ma L, Tian Y, Peng C, Zhang Y, Zhang S (2020). Recent advances in the epidemiology and genetics of acute intermittent porphyria. Intractable Rare Dis Res.

[9] Stenson PD, Mort M, Ball EV, Shaw K, Phillips A, Cooper DN (2014). The Human Gene Mutation Database: building a comprehensive mutation repository for clinical and molecular genetics, diagnostic testing and personalized genomic medicine. Hum Genet.

[10] Andersson C, Floderus Y, Wikberg A, Lithner F (2000). The W198X and R173W mutations in the porphobilinogen deaminase gene in acute intermittent porphyria have higher clinical penetrance than R167W. A population-based study. Scand J Clin Lab Invest.

[11] Elder G, Harper P, Badminton M, Sandberg S, Deybach JC (2013). The incidence of inherited porphyrias in Europe. J Inherit Metab Dis.

[12] Lenglet H, Schmitt C, Grange T (2018). From a dominant to an oligogenic model of inheritance with environmental modifiers in acute intermittent porphyria. Hum Mol Genet.

[13] Demasi M, Penatti CA, DeLucia R, Bechara EJ (1996). The prooxidant effect of 5-aminolevulinic acid in the brain tissue of rats: Implications in neuropsychiatric manifestations in porphyrias. Free Radic Biol Med.

[14] Schutte CM, van der Meyden CH, van Niekerk L, Kakaza M, van Coller R, Ueckermann V, Oosthuizen NM (2015). Severe porphyric neuropathy--importance of screening for porphyria in Guillain-Barré syndrome. S Afr Med J.

[15] Barreda-Sánchez M, Buendía-Martínez J, Glover-López G (2019). High penetrance of acute intermittent porphyria in a Spanish founder mutation population and CYP2D6 genotype as a susceptibility factor. Orphanet J Rare Dis.

[16] Helson L, Braverman S, Mangiardi J (1993). Delta-aminolevulinic acid effects on neuronal and glial tumor cell lines. Neurochem Res.

[17] Wang B (2021). The acute hepatic porphyrias. Transl Gastroenterol Hepatol.

[18] Gandhi Mehta RK, Caress JB, Rudnick SR, Bonkovsky HL (2021). Porphyric neuropathy. Muscle Nerve.

[19] Suarez JI, Cohen ML, Larkin J, Kernich CA, Hricik DE, Daroff RB (1997). Acute intermittent porphyria: clinicopathologic correlation. Report of a case and review of the literature. Neurology.

[20] Pischik E, Kauppinen R (2009). Neurological manifestations of acute intermittent porphyria. Cell Mol Biol (Noisy-le-grand).

[21] Wang B, Rudnick S, Cengia B, Bonkovsky HL (2019). Acute hepatic porphyrias: review and recent progress. Hepatol Commun.

[22] Albers JW, Fink JK (2004). Porphyric neuropathy. Muscle Nerve.

[23] Kazamel M, Desnick RJ, Quigley JG (2020). Correction to: porphyric neuropathy: pathophysiology, diagnosis, and updated management. Curr Neurol Neurosci Rep.

[24] Ponciano A, Carvalho JN, Gala D, Leite J, Fernandes C (2020). Pearls & oy-sters: Guillain-Barré syndrome: an unusual presentation of acute intermittent porphyria. Neurology.

[25] Pischik E, Kauppinen R (2015). An update of clinical management of acute intermittent porphyria. Appl Clin Genet.

[26] (2023). The drug database for acute porphyria. http://www.drugs-porphyria.org/.

[27] Shenhav S, Gemer O, Sassoon E, Segal S (1997). Acute intermittent porphyria precipitated by hyperemesis and metoclopramide treatment in pregnancy. Acta Obstet Gynecol Scand.

[28] Park EY, Kim YS, Lim KJ, Lee HK, Lee SK, Choi H, Kang MH (2014). Severe neurologic manifestations in acute intermittent porphyria developed after spine surgery under general anesthesia: a case report. Korean J Anesthesiol.

[29] Dover SB, Plenderleith L, Moore MR, McColl KE (1994). Safety of general anaesthesia and surgery in acute hepatic porphyria. Gut.

[30] Bissell DM, Anderson KE, Bonkovsky HL (2017). Porphyria. N Engl J Med.

[31] Wylie K, Testai FD (2022). Neurological manifestations of acute porphyrias. Curr Neurol Neurosci Rep.

[32] Syed YY (2021). Givosiran: a review in acute hepatic porphyria. Drugs.

[33] Hinchey J, Chaves C, Appignani B (1996). A reversible posterior leukoencephalopathy syndrome. N Engl J Med.

[34] Zheng X, Liu X, Wang Y (2018). Acute intermittent porphyria presenting with seizures and posterior reversible encephalopathy syndrome: two case reports and a literature review. Medicine (Baltimore).

[35] Lin TC, Lai SL, Hsu SP, Ro LS (2013). Treatment of neuropathic pain in acute intermittent porphyria with gabapentin. J Formos Med Assoc.

[36] Puy H, Gouya L, Deybach JC (2010). Porphyrias. Lancet.

[37] Baumann K, Kauppinen R (2022). Long-term follow-up of acute porphyria in female patients: update of clinical outcome and life expectancy. Mol Genet Metab Rep.

[38] Kaushik R, Kharbanda PS, Bhalla A, Rajan R, Prabhakar S (2014). Acute flaccid paralysis in adults: our experience. J Emerg Trauma Shock.

[39] Jaramillo-Calle DA, Solano JM, Rabinstein AA, Bonkovsky HL (2019). Porphyria-induced posterior reversible encephalopathy syndrome and central nervous system dysfunction. Mol Genet Metab.

[40] Kazamel M, Desnick RJ, Quigley JG (2020). Porphyric neuropathy: pathophysiology, diagnosis, and updated management. Curr Neurol Neurosci Rep.

[41] Bonnefoy Mirralles AM, Torres-Castro R, Ovalle Guzman C (2017). A comprehensive rehabilitation program and follow-up assessment for acute intermittent porphyria. Am J Phys Med Rehabil.

[42] (2020). Fiscal Year (FY) 2021 Inpatient Rehabilitation Facility (IRF) Prospective Payment System (PPS) (CMS-1729-F). https://www.cms.gov/newsroom/fact-sheets/fiscal-year-fy-2021-inpatient-rehabilitation-facility-irf-prospective-payment-system-pps-cms-1729-f.

